# Long-term field performance of a polyester-based long-lasting insecticidal mosquito net in rural Uganda

**DOI:** 10.1186/1475-2875-7-49

**Published:** 2008-03-20

**Authors:** Albert Kilian, Wilson Byamukama, Olivier Pigeon, Francis Atieli, Stephan Duchon, Chi Phan

**Affiliations:** 1 Malaria Consortium, Development House, 56-64 Leonard Street EC2A 4JX, London, UK; 2 Department Health, Education, Social Security, German Technical Cooperation (GTZ), Eschborn, Germany; 3 District Health Services Kabarole District, Fort Portal, Uganda; 4 Department Phytopharmacie, Centre wallon de Recherches agronomiques (CRA-W), Gembloux, Belgium; 5 Division of Parasitic Diseases, Centers for Disease Control and Prevention, Atlanta, USA; 6 Centre for Vector Biology and Control Research, Kenya Medical Research Institute, Kisumu, Kenya; 7 Laboratoire de Lutte contre les Insectes Nuisibles (LIN), Institut de Recherche pour le Developpement (IRD), Montpellier, France; 8 Vestergaard-Frandsen Quality Control Laboratories, Hanoi, Vietnam

## Abstract

**Background:**

In order to evaluate whether criteria for LLIN field performance (phase III) set by the WHO Pesticide Evaluation Scheme are met, first and second generations of one of these products, PermaNet^®^, a polyester net using the coating technology were tested.

**Methods:**

A randomized, double blinded study design was used comparing LLIN to conventionally treated nets and following LLIN for three years under regular household use in rural conditions. Primary outcome measures were deltamethrin residue and bioassay performance (60 minute knock-down and 24 hour mortality after a three minute exposure) using a strain of *Anopheles gambiae s.s*. sensitive to pyrethroid insecticides.

**Results:**

Baseline concentration of deltamethrin was within targets for all net types but was rapidly lost in conventionally treated nets and first generation PermaNet^® ^with median of 0.7 and 2.5 mg/m^2 ^after six months respectively. In contrast, second generation PermaNet^® ^retained insecticide well and had 41.5% of baseline dose after 36 months (28.7 mg/m^2^). Similarly, vector mortality and knockdown dropped to 18% and 70% respectively for first generation LLIN after six months but remained high (88.5% and 97.8% respectively) for second generation PermaNet^® ^after 36 months of follow up at which time 90.0% of nets had either a knockdown rate ≥ 95% or mortality rate ≥ 80%.

**Conclusion:**

Second generation PermaNet^® ^showed excellent results after three years of field use and fulfilled the WHOPES criteria for LLIN. Loss of insecticide on LLIN using coating technology under field conditions was far more influenced by factors associated with handling rather than washing.

## Background

In recent years the use of insecticide treated mosquito nets (ITN) and curtains has been established as one of the key measures of malaria prevention in sub-Sahara Africa with proven efficacy and effectiveness [1]. Attempts are now under way to go to scale with this intervention by involving all possible stakeholders and creating the maximum synergy effects through public-private partnerships. One of the most important obstacles to wide-spread coverage with ITN has been, however, the need for regular re-treatment with insecticide every 6–12 months. Up to date re-treatment rates range from 2% to 20% in most instances and rarely reach more than 40% unless re-treatment is done by the public health services without cost to the consumer [2,3]. The solution to this problem and a true breakthrough in the ITN application is the concept of a long-lasting insecticidal net (LLIN), i.e. a net on which the insecticide effect lasts at least as long as the average useful live of that net even when it is used and washed regularly [4]. Early attempts of increasing wash resistance of permethrin by adding polystyrene to the emulsifiable concentrate [5] showed a significantly prolonged effect but it was not long enough to last for the life of a net.

The new technologies for LLIN can be divided into two types [6]. The first, called incorporation technology, uses polyethylene as material where it is possible to directly incorporate a pyrethroid insecticide into the material from which then the netting is made. Facilitated by certain reagents the insecticide will migrate to the surface of the fibre and will be regenerated from the reservoir after the surface insecticide is washed off or otherwise lost. The second type is based on multifilament polyester as the netting material and here a resin based polymer coating is used as the insecticide reservoir for replacement of surface insecticide and this coating is bound to the surface of the filament. This is called the coating technology.

The first long-lasting insecticidal net (LLIN) based on polyethylene incorporation technology is the Olyset Net^® ^(Sumika Life-Tech Co, Osaka, Japan), pre-treated with permethrin. Effectiveness over at least three years has been shown to be good [7] and based on available evidence this LLIN was recommended for malaria prevention by the WHO Pesticide Evaluation Scheme (WHOPES) in 2001 [8].

Another LLIN based on the coating technology using deltamethrin as insecticide has been developed by Vestergaard Frandsen Disease Control Textiles (VF, Lusanne, Switzerland). This product has been branded PermaNet^®^. Initial field studies showed that these nets had a good level of wash resistance [9]. However, with respect to actual duration of insecticide protection in the field initial data from Burkina Faso seemed to indicate that performance was not quite as good as anticipated although sample sizes for this study were rather small [10].

As a reaction to initial results of varying product performance, the manufacturer reviewed its production processes and reports to have significantly stabilized and improved the manufacturing process. The resulting second generation product was finalized in August 2002 and officially launched in April 2003. It is called PermaNet 2.0^® ^referring to the original product as first generation.

Recent results with later versions of this product [11-13] suggest that indeed the initial problems have been overcome and based on the phase I and II laboratory wash data and experimental hut trials PermaNet 2.0^® ^received a preliminary WHOPES recommendation in 2004 [14]

This study reports on the long-term field performance of the initial as well as the improved, second generation product of PermaNet^® ^as part of the phase III WHOPES evaluation process.

## Methods

### Study area

The study was carried out between 2000 and 2005 in Kyenjojo District in west Uganda, a hilly area with altitudes between 1,350 and 1,550 meters. Annual rainfall ranges from 1,200 to 1,600 mm, mean temperature is 21.3°C, and average relative humidity about 70%. Malaria is meso- to hyperendemic with *Plasmodium falciparum *prevalence rates in asymptomatic children age 2–9 years between 45 % and 68% [15,16]. Main vectors are *Anopheles gambiae *sensu strictu and *An. funestus *with Entomological Inoculation Rates (EIR) estimated around 7 infective bites per person per year [17]. Study site was in Kirongo Parish, Nyantungo Subcounty and involved five villages: Bucuni, Bwendero, Kasunga, Kidomi and Kyakahuli. These had previously participated in a study on insecticide treated curtains showing excellent cooperation. In addition, village health workers with intensive experience in surveys and field work were available.

### Study design and sample size

This was a prospective study with mosquito nets as the unit of observation. Two types of nets, LLIN and ITN, i.e. conventionally treated nets, were randomly distributed to households with users as well as field staff initially blinded with respect to the type of net. In intervals of 6 or 12 months a sample of nets was randomly selected out of the pool of study nets for testing with bioassay (mortality and knockdown rate of mosquitoes) and chemical residue as the principle outcome measures. Two distinct phases of the study can be distinguished each testing a different product of LLIN and comparing it to ITN. Time of follow-up for LLIN was 39 and 36 months respectively and that for ITN was 12 months.

The necessary sample size, i.e. number of nets to be sampled at each time point was calculated based on the main outcome variables. It was found that a sample of 40 nets for each type (LLIN and ITN) would be sufficient. Assuming one measurement for each of the nets per time-point, an alpha error of 0.05, power of 80%, a standard deviation of 8.0 (taken from previous deltamethrin studies) this sample size was sufficient to detect a decline of 8 mg/m^2 ^or more between time points as statistically significant. Similarly, it allows the detection of a difference of at least 15%-points between time points or type of nets in vector knockdown or mortality rates as significant.

While this study was designed before the publication of the WHOPES guidelines for phase III field testing of LLIN [18] its approach is in keeping with these recommendations.

### Tested products and net treatments

Two versions of the LLIN PermaNet^® ^(Vestergaard-Frandsen) were tested. The first generation is a multifilament polyester net treated with a target dose of 50 mg/m^2 ^of deltamethrin using a coating technology to enable wash resistance and create a reservoir of insecticide. The first generation test nets were part of a shipment of 10,000 nets delivered to the Commercial Market Strategies (CMS) project in Kampala in November 2000 for social marketing and were produced in September 2000. Nets were stored in the CMS warehouse in bundles of 50 nets. Nets for the study were identified at random by first selecting 46 bundles from the shelves in the warehouse and then from each bundle 10 nets giving a total of 460 LLIN.

For the second generation PermaNet^® ^no large scale shipment was available and 270 test nets were directly received from Vestergaard-Frandsen in August 2002. These multi-filament polyester nets were part of routine production with a target dose of 55 mg/m^2 ^of deltamethrin. At the time of production (July 2002) the company did not yet use batch numbers on the labels so these are not available.

ITN for phase one (test of first generation PermaNet^®^) were nets identical to the LLIN with respect to netting material but were sent from the factory untreated. The treatment of these 150 nets was done in the CMS warehouse in Kampala using a 1% suspension concentrate of deltamethrin (K-Othrine, Bayer Environmental Science), one of the WHOPES recommended insecticides for net treatment [19]. Based on a surface area of the nets of 13.1 m^2 ^and a target dose of 25 mg/m^2 ^33 ml of insecticide solution and 400 ml of water were used per net. A solution was prepared in a bucket for two nets at a time and nets soaked for two to three minutes under continuous kneading. After excess water had dripped off nets were dried flat on plastic sheeting in the shade of the warehouse.

For the second phase of the study (test of second generation PermaNet^®^) the ITNs comprised one group of the original nets re-treated after 15–18 months of field use and one group of new, multifilament polyester nets (Siamdutch Netting Company, Thailand) which were exchanged for other conventionally treated nets. Net treatments were done in the field using the wetable tablet version of the same WHOPES recommended deltamethrin [19] (KO-Tab, Bayer Environmental Science) containing 360 mg deltamethrin. Nets were treated by experienced and supervised field staff using basins and 400 ml of water and 1 insecticide tablet according to manufacturer's instructions. Nets were dried flat on the grass and as much as possible in the shade.

All nets, LLIN and ITN, were white, rectangular nets of 75 denier and a size of 160 × 150 × 180 cm (width, height, length).

### Field procedures

From each type of net (first and second generation LLIN, ITN) 10 randomly selected nets were kept for baseline analysis while the remaining nets, 450 first generation LLIN, 140 ITN and 260 second generation LLIN were prepared for distribution to households. For the first phase of the study all existing labels on the nets were removed in order to allow blinding and an identification number, printed with wash resistant ink on a piece of polyester band, stitched on the net. The numbers had been previously randomly allocated to the two groups (LLIN and ITN) so that no identification of net type was possible purely by the ID number. After the ID number labels had been fixed to the nets they were re-sorted by ID number thereby mixing the nets at random. Based on a household list from the five villages net numbers were randomly allocated to households according to available sleeping places to ensure equitable distribution. No specific instructions regarding use or washing were given to the net users.

The procedure for second generation PermaNet^® ^was similar with the difference that labels were not removed since at the time only LLIN were distributed. Random allocation to households was done based on "vacancies" according to the master list of nets which was kept by the principle investigator and recorded the status of each net (type, ID number, household number, time of distribution, time of sampling, replacement or removal).

Jointly with the initial net distribution a household survey of all 294 participating households was carried out in December 2000 by experienced field staff following one day of training on the specific questionnaire. In addition to the number of persons and children living in the household the pre-coded questionnaire included information on education and occupation of head of household, physical condition of the house (roof, windows, wall materials, and eaves), family assets and ownership of animals and land.

Between 11 and 15 days after the net distribution a random sample of 116 households was re-visited and a short questionnaire designed to capture any adverse effects of the insecticide on the net users, their duration and severity.

Throughout the study net surveys were carried out among households to collect data from all active study nets on net use (who and frequency of use), perception of net effect, washing habits (method of washing, type of water and soap used) and number of washes since last survey. The physical condition of each study net was assessed with respect to where the net was found (hanging or not) and number, size and position of holes. Three categories of hole sizes were used: up to the diameter or length of a coin, hand width or larger than a hand width. To mark the position of a hole the net was divided into numbered areas (upper and lower part of each side) and the code for the hole's location entered into the questionnaire. A detailed list of the time intervals of the surveys and the number of nets seen is given in the annex [see Additional file 1]. A total of 13 surveys were carried out for phase one nets and seven for phase two nets. For logistic and operational reasons the frequency of surveys per year reduced over time from four in the first, three each in the second and third and two each in the fourth and fifth. During the course of the study the questionnaire for net follow-up was simplified as the information obtained did not change over time. The first change was made in April 2002 (survey 5) when assessment of the hole's position and the information on who used the net were ended and the second in October 2004 (survey 11) when questions on the method of washing and net perception were ended.

### Sampling and sample preparation

For each time point a random sample of 40 nets per net type were selected from the net master list together with 2 possible replacements in case the selected households could not be reached or the net had been lost to follow-up since the last visit. Nets were then collected by the field team and each household received a LLIN as a replacement to insure continuous protection of the family. These replacements nets, however, did not have ID numbers and were not included in net follow-up surveys. Details of collected samples at various time points are presented in the annex [see Additional file 2].

Using templates of 30 × 30 cm (bioassay) and 10 × 10 cm (chemical residue) samples were cut out of the nets, marked with the date and the net ID number, packed in aluminium foil and stored at room temperature until transport to the respective laboratories for analysis. Samples for bioassay and chemical residue were always taken from the same spot (long side near the ID number mid-way between top and bottom) immediately next to each other. Generally one sample was taken per net per time point with the following exceptions in order to allow assessment of intra- and inter-net variability. At baseline and 6 months of the first phase a second sample was taken from the short side of the net for bioassay and chemical residue. These locations were termed sites one (standard sample) and two respectively. At base line of the first phase two additional samples for chemical residue were taken from each site immediately next to each other and these were termed position one and two respectively. Therefore, there were 2 samples (site 1 and 2) per net for bioassay at baseline and 6 months while for chemical residue there were 4 samples at baseline (position 1 and 2 each for site 1 and 2) and 2 at 6 months (site 1 and 2). For the second phase additional samples were only taken at baseline and only for chemical residue (site 1 and 2).

After the first samples had been taken the ten baseline nets for the first generation LLIN were kept outside the package exposed to air and dust but not sun and were not used or washed. Samples from these nets were taken 11, 27, 39 and 60 months after the unpacking and sent for chemical and bioassay analysis.

### Chemical residue

All samples were analysed at the laboratory of the Pesticides Research Department of the Walloon Agricultural Research Centre in Gembloux, Belgium (WHO Collaborating Centre) using the MEREPERMA methodology which has been ISO accredited (ISO 17025). Surface area and weight of each 10 × 10 cm sample was measured and the sample then introduced into a 100 mL Erlenmeyer flask. Deltamethrin was extracted from the sample by heating under reflux for 60 minutes with 40 mL xylene. After cooling to ambient temperature the extract was quantitatively transferred into a 50 mL volumetric flask. The flask was filled up to volume with xylene. A 10 times dilution was achieved in xylene. The final extract was then analysed for determination of deltamethrin by Capillary Gas Chromatography with ^63^Ni Electron Capture Detection (GC-ECD) using an external standard calibration. For each sample two chromatographic injections were performed and the mean reported as g/kg deltamethrin and then transformed to mg/m^2 ^based on the surface area of that sample. The analytical method was validated for the determination of deltamethrin residues in conventionally and long lasting treated nets. Specificity, repeatability (precision), linearity of the detector response, recoveries (accuracy) and limit of quantification were determined. The accuracy of the method was determined concurrently with the analysis of samples from 2001 to 2005 by spiking untreated mosquito net samples (which had already been extracted) with know amounts of deltamethrin. The mean recovery varied between 95% and 101 % depending on level of deltamethrin concentration (n = 242) with a Relative Standard Deviation (RSD) between 7% and 11% for fortification levels ranging from 0.3 mg/m^2 ^to 100 mg/m^2^. The acceptable limit is 90–110 % with a RSD < 15 %. Therefore, the accuracy and precision of the analytical method was found to be excellent.

A total of 115 samples from both first and second phase of the study which had been taken immediately next to the sample for GC-ECD analysis were also analysed at the Vestergaard-Frandsen Quality Control Laboratories in Hanoi, Vietnam using a method where insecticide determination is done by normal phase High Performance Liquid Chromatography with UV Diode Array Detection (HPLC-DAD) using an internal standard. The principle of this method was proposed in 2006 for adoption by the Collaborative International Pesticides Analytical Council (CIPAC). In brief, net samples are cut into small pieces of < 2 × 2 cm and deltamethrin is extracted into solution using a mixture of solvents iso-octan plus 1,4 dioxan with 0.15% HPLC grade water (80/20, v/v). Dibutyl phthalate is added as the internal standard. The extraction bottle is sonicated in a water bath set at 80°C and then shaken vigorously for at least 15 minutes. A proper volume of solution is filtered through 0.45 micrometer membrane syringe filter into a vial. A volume of 5 μL of filtered solution is injected into a normal phase isocratic HPLC equipped with PDA/UV detector and deltamethrin is quantified using an internal standard calibration curve. The method was shown to be suitable for deltamethrin with repeatability (same net sample) of 1.8% (RSD, n = 7), reproducibility (multiple samples over time) of 11.6% (RSD) and recovery of 99.7% (95% CI 98.6%–101.6%) from samples in which deltamethrin content was added at an exact amount by weighting method (n = 5).

### Bioassays

Bioassays for the first study phase were carried out by the Laboratoire de Lutte contre les Insectes Nuisible, Montpellier, France (WHO Collaborating Centre) using WHO standardized procedures [20]. For the tests 2–4 day old, unfed female *Anopheles gambiae s.s*.(Kisumu strain) and *Culex quinquefasciatus *(S-lab strain) were used, the latter were only tested for the first 12 months. Both species have been well established in culture and are known to be pyrethroid sensitive. The tests were conducted using the standard WHO plastic cones and a three minute exposure time. Five mosquitoes were introduced into cones at a time. Tests were carried out at 25°C ± 2 under subdued light. After exposure, females were grouped into batches of 10 or 20 in 200 mL plastic cups and maintained at 28°C ± 2 and 80% ± 10% relative humidity with honey solution provided. For each sample tested, a total of 50 mosquitoes were used (Inter Quartile Range 50–51, range 40–62, ten cones). Proportion of mosquitoes knocked down at 60 minutes (KD60) was calculated. Percentage mortalities were recorded after 24 h.

Bioassays for the second phase of the study were carried out at the entomology laboratories at Centers for Disease Control (CDC), Atlanta, USA (WHO Collaborating Centre). Tests were carried out with *Anopheles gambiae s.s*. Exposure time and method were identical, however, only 40 mosquitoes were used per test.

For all bioassays unexposed controls were run to validate the tests results and results were discarded if mortality among control was > 5%.

The definitions of effectiveness of nets based on bioassay results followed recommendations by WHO (Pierre Guillet, personal communication) and were as follows:

Minimal effectiveness: KD60 ≥ 75% or functional mortality ≥ 50%

Optimal effectiveness: KD60 ≥ 95% or functional mortality ≥ 80%

### Data analysis

Data were entered using EpiInfo 6.04 software (WHO/CDC, 1997) and then transferred to Stata 8.2 (Stata Corporation, Texas, USA, 2005) for further data management and analysis.

From the household data a socio-economic index was calculated using principal component analysis considering education, ratio household members per bed, physical condition of house, assets (radio, vehicles), animals and land possession. Only the first component was used to build the index. Households were then divided into wealth quintiles for further analysis.

After appropriate data preparation cumulative washes per net were calculated until the net was censured, i.e. sampled or otherwise lost to follow-up. Similarly, a hole index was calculated for each net and time point which was constructed by multiplying the number of holes with the hole category (1–3 and increasing with size) and then calculating a mean over all nets in the sample, including those with no holes.

For the analysis of net performance (chemical residue, bioassay) the mean of all samples per net was calculated. For the expression of the central tendency of sample measurements of all outcome variables mean, geometric mean or median was chosen after evaluation of the distribution of values within the sample.

For the assessment of between-net variability of insecticide concentration the standard deviation of deltamethrin residue was expressed as percent of the sample mean (coefficient of variation). Intra-net variability was expressed as the difference of samples of the same net to the mean expressed as percent of the mean and then averaged over the sample. For the analysis of statistical differences of inter-intra net variations of outcomes ANOVA was used.

Statistical analysis was generally done in two steps, first univariate analysis was carried out considering all co-variables of interest and tested using Chi-squared test for categorical and t-test or Kruskal-Wallis test for continuous variables depending on the validity of the assumption of normal distribution of values. In a second step multivariate analysis (linear or logistic regression models as appropriate) was used to verify any associations found in the univariate analysis.

### Ethical considerations and approval

This study was conducted according to the principles of the Declaration of Helsinki and the international guidelines of biomedical research involving human subjects. It was reviewed and approved by the Ministry of Health, Uganda, WHO Roll Back Malaria Project, Geneva and Gesellschaft für Technische Zusammenarbeit, GTZ, Germany.

## Results

### Household characteristics

The 590 nets of phase one were initially distributed among 294 households. In the course of the study a total of 31 households dropped out. In 16 cases (5.4% of all households) the reason was that the head of household had died and the family moved away, 10 households (3.4%) shifted to another village and 5 households (1.7%) decided they did no longer want to participate in the study. The study nets of these households were given to other families within the same village.

The majority of heads of households were male while 19.5% were female. Female heads of household were on average older than males (mean 49.0 years versus 37.5 in males), were more likely to be farmers rather than businesspersons (87.7% subsistence farmer versus 65.3% among males). Female heads of household also had less education with 57.9% illiterate, 24.6% primary and 21.1% secondary education while the respective figures for male heads of household were 20.8%, 41.3% and 37.7%.

Of the 294 houses 69.4% had roofs made of corrugated iron while the rest had thatched roofs, 54.6% had plastered walls, 44.7% mudded and in 2 houses (0.7%) the walls were made of thatch. In 56.4% of houses windows could be closed while the remainder were open and 42.8% had an open space (eave) between walls and roof. Only 15 houses (5.1%) had a fire place within the house.

The total population in the study households was 1,661 persons (mean 5.6 per household) and 747 children less than 10 years of age (mean 2.5 per household). The mean number of persons per bed or sleeping place was 2.4. While the number of persons (4.7 to 6.5) as well as children (2.2 to 2.8) per household continuously and significantly increased from the lowest to the highest wealth quintile (both p < 0.01 linear regression) the number of persons per bed decreased with increasing wealth quintile from 2.8 to 2.1 per bed (p < 0.0001).

### Net distribution, side effects, use, washing and physical condition

#### Distribution

The distribution of nets between villages and the proportion of conventionally treated nets and LLIN in each village was very homogeneous reflecting the random distribution. In the first phase the overall proportion of LLIN was 76.3% (450/590) and the proportion of LLIN in each of the villages varied between 73.1% and 80.5% (p = 0.6 Chi Squared). Similarly, the distribution of LLIN between villages in the second phase was identical with that of the first phase (p = 0.9 Chi Squared). The proportion of households that had received only LLIN was 59.7% while 8.9% had only conventionally treated nets and 31.4% had received a mix of net types. This distribution pattern did not differ between villages (p = 0.7 Chi Squared). The mean number of nets per household was 2.0 with 27% of households receiving 1 net, 48% 2 nets, 22% 3 nets and 3% 4 nets. Another evidence for the random distribution and selection of nets is the finding that the mean months of observation per net (23.6) was very much the same in all the villages with a range of 23.0 to 23.8 and p = 0.97 (t-test).

#### Adverse effects

The 116 households included in the assessment of early adverse effects represented 233 nets (39.5% of all nets) and the distribution of nets per household as well as the proportion of ITN and LLIN was not different in this sub-sample compared to the overall study. All respondents confirmed they had used the nets during the first four days after receiving them and 98.3% had used them on all four days. One household had not used the nets on days 2 and 3 because the family had been away and a second household reported to have folded the nets away on days 3 and 4 due to adverse effects. A total of 15 households (12.9% 95% CI 7.4–20.4) reported adverse effects during the first 4 days of utilization. In 9 cases (7.8%) irritations of nose, eyes or skin were reported and in 6 cases (5.2%) the effect was described as "feeling heat". There was a clear relationship between reported adverse effects and the time between unpacking of nets at the district offices and distribution to the households: 48% of households that received the nets within four days after unpacking reporting any effects but only 7% of households that received the nets 5–7 days after unpacking and none if the distribution occurred between 8 and 10 days after unpacking. In a logistic regression model the odds of reporting any side effects reduced by 40% per day after unpacking (OR 0.61, p < 0.001). The same was true for the duration of the adverse effects. In 10 households (67% of those with any adverse reactions) the effects lasted for only 1 or 2 days, 1 household (7%) reported duration of 3 days and in 4 households (27%) they lasted for 4 or more days. All of the latter came from the village were nets had been distributed immediately after unpacking. Finally, adverse effects were analysed separately for households with only LLIN, only ITN or both. In households with only ITNs 22% reported side effects, 18% in those with both and 9% in households that only had LLIN. However, the difference was not statistically significant (p = 0.17 after adjustment for time since distribution, logistic regression).

#### Net use

Details of all net surveys undertaken and the times and numbers of nets sampled, exchanged or retreated are presented in the annex [see Additional file 1 and 2]. In total only 43 nets (5.1%) were lost to follow-up. Due to the continuous sampling of nets the sample size reduced over time. The median number of times nets were seen in the first study phase was 8 (Inter-Quartile Range 6–11) with 53 nets being seen at all 13 surveys. For the second phase the median number of observations was 5 (IQR 4–7) and 62 nets were seen at all 7 surveys. In total 5,199 observations on nets were recorded, 4,020 on phase one nets and 1,179 from the second phase.

The vast majority of nets (96.9%) were found hanging over the sleeping place at the time of the survey while 2.7% were stored away and 0.4% were not present on that day. Among those hanging over the sleeping place 82.1% were folded up while 17.9% were hanging in "sleeping position". However, changes in hanging patterns could be detected over time. At net age of 2 years or less 2.0% of nets were stored and this rate increased to 3.5% between 2–3 years, 5.5% between 3–4 years and 12.0% after 4–5 years. This trend was statistically significant (p < 0.0001, non-parametric test for trend). Similarly, the proportion of nets hanging openly over the bed or mattress among those hanging decreased with increasing age of net: from 19.5% after 2 years or less to 13.3% at 2–3 years, 12.9% at 3–4 years and 5.0% at 4–5 years (p < 0.001). After adjusting for age of net no differences in hanging patterns were found between the net types.

Daily use in the past week was reported for 94.4% of net observations while 2.9% had been used more than half of the days, 1.7% less than half of the days and 1.0% did not know. Net use did not vary between dry and rainy season and only decreased slightly at net age 4–5 years when 8.4% of nets were not used every night. Again no major differences were found between net types.

The sleeping patterns, i.e. which family members used the net were only assessed during the first study phase until survey 10 (37 months after start). Overall in 17.1% of net observations the net was reported to have been used only by adults, in 38.7% by an adult and child and in 44.2% only by children. Interestingly, children under 5 years of age generally slept with an adult and only in 4.3% of net observations did a child under 5 sleep alone or with older siblings. The reported sleeping patterns varied somewhat from survey to survey but without major trends. The only slight change over time was a shift from adults sleeping with children to children alone. The former decreased from 40.0% to 31.7% while the latter increased from 43.7% to 50.4%. The data for phase two nets (only first 24 months) were very similar with only a slight increase in the proportion of "adults alone" to 20.6%.

Questions regarding the perception of the nets by the user did not produce a distinction between ITN and LLIN and tended to be 100% approval after the first few surveys. This was the case with questions on whether net users felt negatively affected by the nets (too hot or too cold, closed in) as well as with questions asking for the perceived usefulness of the net against mosquitoes and febrile illness. There also was no difference between the first and the second phase of the study and these questions were ended with survey 10.

#### Washing

Washing of nets was done in a basin using cold water and country soap. There were only few exceptions with two out of 2,510 washes reported to have been done with hot water and in 11 washes a branded detergent (OMO, NUOMI etc) was used. The use of such detergents was only reported in the first 3 surveys of phase one and showed a declining rate: 3.6% survey 1, 2.5% survey 2 and 0.5% survey 3. Eight of the 11 detergent washes occurred in 3 neighbouring households in Kyakahuli village. Alkalinity of the country soap bars was found to be lower (ph 9–10) than that of the detergent (ph 11–12) when a saturated soap solution was tested with Litmus paper strips.

**Figure 1:**
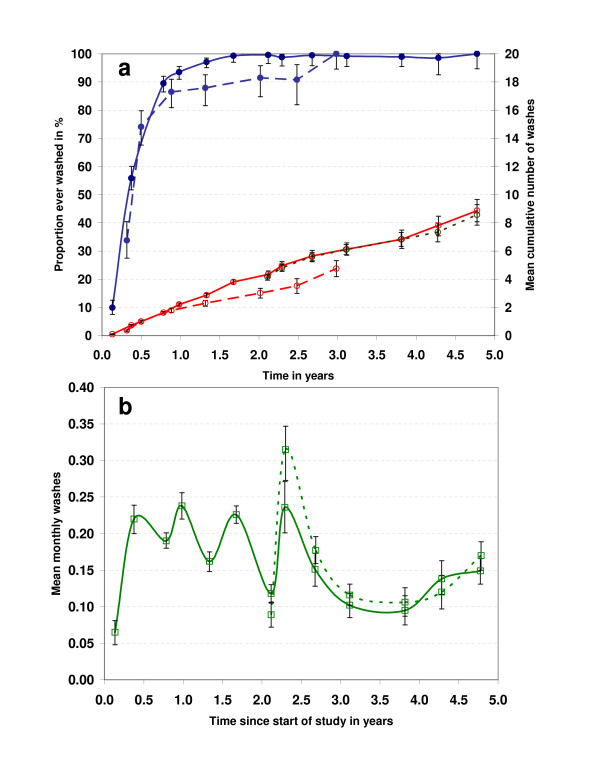
**Washing: Proportion ever washed, mean washes and wash frequency**. **a**: Proportion of nets ever washed (filled circles) and mean number of washes (open circles) for phase one (line) and phase two (dashed). Error bars represent 95% confidence intervals. The dotted line (open circles) shows the mean number of washes for phase 2 nets when aligned in time since start of study rather than time of observation. **b**: Mean monthly washes between surveys for phase one (line) and phase 2 (dotted). Error bars represent 95% confidence intervals.

Figure 1a shows the proportion of nets ever reported washed and the mean number of cumulative washes as a function of time of observation. In both phases of the study a rapid increase in the proportion of nets washed at least once was observed reaching 85% to 95% after one year. However, time to > 98% washing saturation was longer in phase 2 with 7/76 nets observed for 30 month not reported ever washed compared to 1/139 after 32 months during phase one. Accordingly, median time to first wash was a bit shorter in phase one (4.6 months, no difference between ITN and LLIN) than phase two (6.0 months) but the difference was not statistically significant (p = 0.8, Kruskal-Wallis test). Mean number of washes per net and year was 2.2 (95% CI 2.1–2.3) during phase one with no difference between LLIN and ITN (p = 0.4, t-test). During phase two washing frequency was significantly lower with 1.8 (95% CI 1.7–1.9) washes per net per year. Therefore, cumulative washes for second generation LLIN (phase two) in Figure 1a appear lower than first generation LLIN and ITN as they are plotted against time of observation. However, when the curve for second generation LLIN is shifted so that it is synchronized with phase one nets in time since start of study (short dashed line in Figure 1a) it becomes evident that there was no difference between net types during phase two. Mean cumulative washes after 3 years of use was 6.0 in phase one and 4.8 in phase two with a maximum of 11 and 9 washes respectively.

The decline of washing frequency over time without major differences between the two study phases and net types can also be seen in Figure 1b which shows the mean monthly washings in the inter-survey intervals. Interestingly, there is a clear and statistically significant seasonal variation within the first 2 years when 4 surveys were done per year. Survey intervals that covered the rainy seasons of March to May and September to December showed higher washing frequency than those covering the dry season [see Additional file 1 for survey dates]. This seasonality is not visible when survey frequency was only 3 or 2 per year during phase two. While washing frequency did not differ with wealth quintile in this population it did vary significantly between villages (p < 0.0001, ANOVA) with mean annual washes ranging from 1.7 to 2.7. Washing frequency declined from first to second phase in all villages but one which remained at a mean of 2.4 washes per net per year but was strongest in the two villages which in the first phase showed the lowest washing rates (decline from 2.2 to 1.1).

Table 1 shows the mean number of washes of the nets sampled for testing. Mean washes per net as well as range of washes does not differ from the values obtained from the surveys of the total net population and after 36–39 months is, accordingly, higher for phase one nets than for phase two nets.

**Table 1:** Washes of net samples. PN1 = Permanet 1^st ^generation, PN2 = Permanet 2^nd ^generation, conv = conventionally treated.

**Months since start**	**Wash frequency of sampled nets **Mean (95% CI) Range		
	**PN1**	**Conv**	**PN2**

3		0.4 (0.1–0.7) 0–1	
6	0.7 (0.5–1.0) 0–3	0.9 (0.7–1.0) 0–3	0.9 (0.5–1.2) 0–4
12	2.2 (1.8–2.5) 0–4	1.5 (0.8–2.3) 0–3	2.0 (1.5–2.4) 0–4
18			2.4 (2.0–2.9) 0–5
20	3.8 (3.3–4.3) 1–8		
24			3.0 (2.4–3.6) 0–7
27	4.7 (4.0–5.3) 1–8		
36			4.5 (3.7–5.2) 0–9
39	6.1 (5.3–6.9) 2–11		

#### Physical conditions

**Figure 2:**
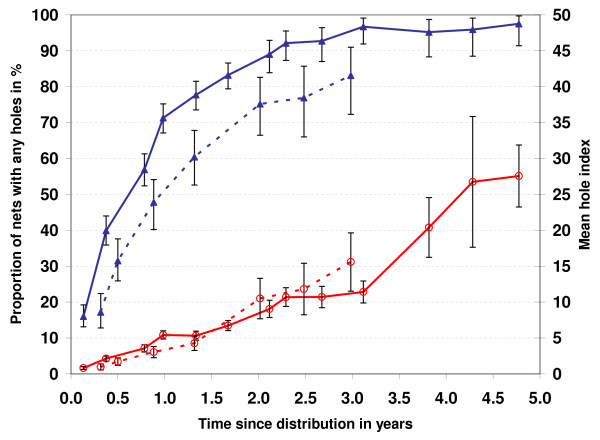
**Physical condition of nets**. Proportion of nets with any holes (filled triangles) and mean hole index (open circles) for phase one (line) and phase two (dashed). Error bars represent 95% confidence intervals.

The proportion of nets found to have any holes is shown in Figure 2. There was a rapid increase in the proportion of the nets found with holes during the first year of the life of the net. During phase one more than 70% of nets had holes after only one year and more than 85% after 2 years. ITN tended to have a lower hole rate but the difference was only statistically significant between surveys 4 and 6. The increase of the proportion of nets with any holes over time was similar in shape during phase two of the study but the curve was shifted downwards with all time points except the first significantly lower than during phase one and less than 50% of nets showing holes after one year.

The hole index captures not only the presence or absence of holes but also takes into account number and size (see methods for details of calculation). As shown in Figure 2 the mean hole index increased linearly during the first 3 years of net follow up with a mean of 4.4 (95% CI 3.8–4.5) after one year (all nets combined), 7.5 (6.7–8.4) after two years and 12.4 (10.7–14.1) after 3 years. After three years it appears to increase more rapidly reaching 20.4 (16.2–24.6) after four and 27.6 (23.2–31.9) after almost five years. This trend of accelerating decay of the nets after 2–3 years was even more evident when the proportion of nets with a hole index above 30 was calculated. While this proportion was 0 or less than 1% until year 2 it increased to 2.3%, 6.7%, 22.6%, and 44.4% at 2, 3, 4 and 5 years of follow up.

In order to see whether the physical condition of the net had any impact on net utilization the information on whether or not the net was found hanging over the bed or stored away at the time of the surveys was used. The proportion found stored away was 1.7% (95% CI 1.4–2.2) when the hole index was less than 30 and 11.8% (6.6–19.0) when the hole index was 31 or higher resulting in a risk ratio of 6.6 (95% CI 3.9–11.1) and p < 0.00001 (Chi-squared). This association remained unchanged when controlled for time of observation or type of net.

Other factors found to be associated with the change of physical condition over time was the number of washes and socio-economic status. In a linear regression model controlling for the time of observation for the net the maximum hole index observed increased by 1.0 with each wash (p = 0.001) and was on average 3.4 lower in the third and forth highest wealth quintile (p = 0.003). Interestingly, the hole index increased again in the highest wealth quintile although it remained unclear why that was so. No difference in the hole index was found in the regression model between net types.

The location of holes on the net was explored during the first 12 months of phase one. For up to 9 holes per net the location of the hole was noted according to the sections of the net (see methods) with a total of 1,471 hole positions recorded. Three quarters (75.6%) of the holes were found at the lower half of the net where it was touching the bed frame or tucked under the mattress mostly not more than 30 cm from the bottom, 13.6% of holes at the upper part of the net, usually close to the roof and 10.7% on the roof itself. Holes were found at about the same rate at the four sides of the net. From observation of the holes three distinct categories could be distinguished. The most common were holes of longitudinal shape mainly found at the bottom and the top corners followed by round holes either through burning or ripping and the third category were holes with fuzzy edges which most likely were caused by rodents. The latter holes could get quite large and were found on the roof as well as on the bottom of the net.

### Performance of nets

Table 2a presents the results of the chemical residue of deltamethrin by type of net and time of follow-up. While at baseline the distribution of values was in keeping with a normal distribution this was not the case at later points in time and, accordingly, the median was chosen as the measure of central tendency. The median deltamethrin concentration for the first generation PermaNet^® ^at the start of the study was 47.5 mg/m^2 ^(four samples per net) and 69.2 mg/m^2 ^(two samples per net) for the second generation. The respective means were 43.6 mg/m^2 ^(95% CI 36.0–51.3; range 27.6–56.2) and 67.1 mg/m^2 ^(95% CI 60.0–74.4; range 52.5–80.4), respectively. When the content was expressed in g/kg, the units of the actual measurement, the baseline mean for the first generation PermaNet^® ^was 1.5 g/kg (95% CI 1.3–1.7; range 0.9–2.0) and for the second generation 2.1 g/kg (95% CI 1.9–2.4; range 1.5–2.5). As the transformation of g/kg to mg/m^2 ^was based on the measured weight per surface area of each sample, which was on average 29.5 g/m^2 ^for the first and 31.7 g/m^2 ^for the second generation PermaNet^®^, these results deviate slightly from what would have been calculated using the standard 30 g/m^2^. Baseline measurements of deltamethrin on the ITN gave a median of 16.9 mg/m^2 ^and mean 20.1 mg/m^2 ^(95% CI 10.5–29.7; range 6.0–34.5). In contrast, ITN retreated after 18 months and serving as baseline for phase two of the study showed very high variability of content ranging from 1.7 mg/m^2 ^to 87.0 mg/m^2 ^with a median of 42.8 mg/m^2 ^and mean 43.4 (95% CI 23.5–63.1).

**Table 2:** Results from chemical residue analysis. Median deltamethrin residue (in mg/m^2^) and the proportion of nets with a deltamethrin concentration of at least 4 mg/m^2^.

**A**	**Average net chemical residue in mg/m^2 ^at different times of follow-up**					
**Type of net**	**Baseline (n) Median *IQR***	**6 Months (n) Median *IQR***	**12 Months (n) Median *IQR***	**18/20 Months (n) Median *IQR***	**24/27 Months (n) Median *IQR***	**36/39 Months (n) Median *IQR***

PermaNet 1^st ^generation	(10) **47.5** *32.9 – 52.2*	(40) **2.5** *1. 6 – 7.1*	(40) **2.1** *0.7 – 3.9*	(43) **1.0** *0.5 – 2.8*	(40) **1.6** *0.8 – 3.3*	(38) **0.5** *0.3 – 0.9*
PermaNet 2^nd ^generation	(10) **69.2** *56.8 – 75.4*	(40) **65.6** *58.1 – 78.5*	(40) **55.8** *39.1 – 82.4*	(40) **44.5** *34.5 – 67.4*	(38) **32.3** *21.4 – 48.7*	(40) **28.7** *11.2 – 37.9*
Conventional net	(10) **16.9** *10.0 – 25.4*	(40) **0.7** *0.4 – 1.4*	Not done	Not done	Not done	Not done
Conventional net retreated	(13) **42.8** *12.5 – 77.1*	(34) **3.1** *1.6 – 11.5*	(11) **1.4** *0.3 – 26.4*	Not done	Not done	Not done

**B**	**Proportion of nets with deltamethrin ≥ 4 mg/m^2 ^at different times of follow-up**					

**Type of net**	**Baseline (n) % *95% CI***	**6 Months (n) % *95% CI***	**12 Months (n) % *95% CI***	**18/20 Months (n) % *95% CI***	**24/27 Months (n) % *95% CI***	**36/39 Months (n) % *95% CI***

PermaNet 1^st ^generation	(10) **100** *66.2 – 100*	(40) **40.0** *24.9 – 56.7*	(40) **22.5** *10.8 – 38.5*	(43) **15.0** *5.6 – 29.8*	(40) **17.5** *7.3 – 32.8*	(38) **5.3** *0.1 – 17.7*
PermaNet 2^nd ^generation	(10) **100** *66.2 – 100*	(40) **100** *91.2 – 100*	(40) **100** *91.2 – 100*	(40) **100** *91.2 – 100*	(38) **97.4** *86.2 – 99.9*	(40) **90.0** *76.3 – 97.2*
Conventional net	(10) **100** *66.2 – 100*	(40) **5.0** *0.6 – 16.9*	Not done	Not done	Not done	Not done
Conventional net retreated	(13) **100** *75.3 – 100*	(34) **47.1** *29.8 -64.9*	(11) **45.5** *16.7 – 76.6*	Not done	Not done	Not done

**Figure 3:**
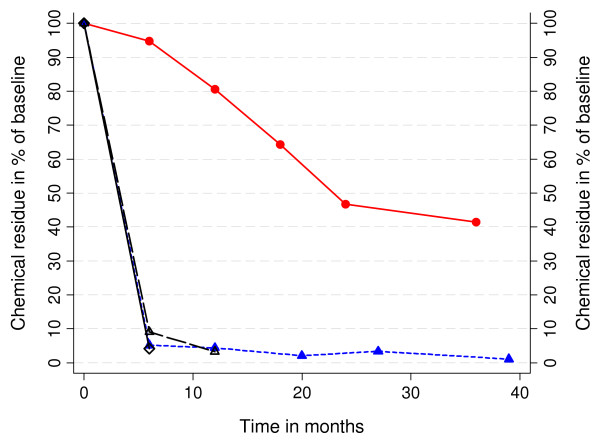
**Decline of chemical residue of deltamethrin as percentage of baseline**. Filled triangle (blue) = first generation LLIN, filled circles (red) second generation LLIN, open diamond = conventional ITN, open triangle = conventional ITN retreated.

Surprisingly, the deltamethrin concentration not only of ITN but also of the first generation PermaNet^® ^dropped dramatically after 6 months of field use to around 5% of the original content (Figure 3). In contrast, decline of deltamethrin was very gradual for the second generation PermaNet^® ^with 41.5 % still left after three years of field use and a median deltamethrin concentration (28.7 mg/m^2^) in the range of the target dose for conventionally treated nets. Interestingly, the deltamethrin content on first generation LLIN did not drop further after six months but remained more or less constant around 2 mg/m^2 ^until 39 months of observation with about one fifth of the sampled nets at each time point having more than 4 mg/m^2 ^deltamethrin (Table 2b). ITN at six months for phase two comprised of a mix of nets: 10 new nets (freshly treated) that had been included as response to the rapid decline of insecticide in the first batch of conventional nets, 10 previous control nets retreated after 15 months and 14 previous control nets retreated after 18 months (together with the second "baseline" nets). While the first two groups showed very similar results (median 2.0 mg/m^2 ^and 2.6 mg/m^2 ^deltamethrin respectively) the third group continued to show slightly higher values with a median of 11.6 mg/m^2. ^However, these differences were not statistically significant.

Results from bioassays with *Anopheles gambiae s.s*. are presented in Table 3. As can be expected, the drop in functional mortality over time (Table 3a) was more rapid and more pronounced than the 60 minute knockdown rates but both variables showed a pattern very similar to what was described for the chemical analysis: first generation LLINs showed a sharp decline after only 6 months with both functional mortality (18.1%) and knockdown rate (69.9%) significantly below the values of 80% and 95% respectively requested by WHOPES but then maintained that level until 39 months of follow up. Results of first generation PermaNet^® ^were significantly better compared to the original ITN but not compared to the second group. In contrast, mortality as well as knockdown rates for the second generation PermaNet^® ^were above the WHOPES cut-off at all time points except for the 24 months values which were slightly below (73.9% mortality, 92.4% knockdown) but in both cases the confidence interval included the cut-off value. Also, three of the five nets with poor results in the bioassay at 24 months still had deltamethrin concentrations above 4 mg/m^2 ^and had been washed immediately before collection. ITN for phase two showed significantly better results than for phase one with the same pattern described for chemical residue: the 14 nets retreated at 18 months showed better results than the 10 new nets and those 10 retreated at 15 months but without reaching statistical significance level.

**Table 3:** Bioassay data with *Anopheles gambiae *s.s (Kisumu strain). Three minute exposure using the cone test. GM = geometric mean, CI = confidence interval.

**A**	**Average net functional mortality in % at different times of follow-up**					
**Type of net**	**Baseline (n) GM *95% CI***	**6 Months (n) GM *95% CI***	**12 Months (n) GM *95% CI***	**18/20 Months (n) GM *95% CI***	**24/27 Months (n) GM *95% CI***	**36/39 Months (n) GM *95% CI***

PermaNet 1^st ^generation	(10) **96.9** *95.3 – 98.6*	(40) **18.1** *13.5 – 24.2*	(40) **15.1** *9.8 – 23.3*	(40) **8.9** *5.6 – 14.1*	(40) **21.1** *14.5 – 30.5*	(38) **9.7** *5.3 – 17.9*
PermaNet 2^nd ^generation	(10) **99.8** *99.3 – 100*	(40) **99.5** *99.2 – 99.9*	(40) **95.2** *89.2 – 100*	(40) **96.0** *93.5 – 98.6*	(38) **73.9** *61.7 – 88.6*	(40) **88.5** *83.7 – 93.7*
Conventional net	(10) **99.2** *97.5 – 100*	(40) **8.5** *5.9 – 12.2*	Not done	Not done	Not done	Not done
Conventional net retreated	(13) **99.4** *98.7 – 100*	(34) **40.8** *27.6 -60.4*	(11) **44.8** *21.6 – 92.9*	Not done	Not done	Not done

**B**	**Average net knockdown rate within 60 minutes in % at different times of follow-up**					

**Type of net**	**Baseline (n) GM *95% CI***	**6 Months (n) GM *95% CI***	**12 Months (n) GM *95% CI***	**18/20 Months (n) GM *95% CI***	**24/27 Months (n) GM *95% CI***	**36/39 Months (n) GM *95% CI***

PermaNet 1^st ^generation	(10) **100** *100 – 100*	(40) **69.9** *62.9 – 77.7*	(40) **59.3** *42.2 – 77.8*	(40) **23.6** *14.9 – 37.4*	(40) **70.3** *57.0 – 86.7*	(38) **37.4** *24.3 – 57.5*
PermaNet 2^nd ^generation	(10) **100** *100 – 100*	(40) **99.8** *99.5 – 100*	(40) **99.0** *97.9 – 100*	(40) **97.6** *96.0 – 99.3*	(38) **92.4** *85.8 – 99.5*	(40) **97.8** *96.6 – 99.0*
Conventional net	(10) **100** *100 – 100*	(40) **31.6** *24.9 – 41.0*	Not done	Not done	Not done	Not done
Conventional net retreated	(13) **99.8** *99.4 – 100*	(34) **76.9** *62.8 -94.2*	(11) **59.5** *29.0 – 100*	Not done	Not done	Not done

Bioassay results for *Culex quinquefasciatus *were significantly poorer than those for *Anopheles gambiae s.s*. At baseline functional mortality was 80.8% (95% CI 74.6–87.0) with no difference between first generation LLIN and ITN. Values then dropped to 8.1% (3.5–12.7) for LLIN and 2.4% (0.6–4.3) for ITN at 6 months and 4.9% (1.6–8.2) for LLIN at 12 months. Knockdown results were similar with 100% at baseline, 16.0% (7.5–24.5) LLIN and 4.6% (0.9–8.3) ITN at 6 months and 22.5% (12.4–32.6) LLIN at 12 months.

Combining mosquito mortality and knockdown the minimal and optimal effectiveness was calculated and is presented in Table 4. After 36 months 90.0% of second generation PermaNet^® ^still had optimal effectiveness with either a knockdown rate ≥ 95% or mortality rate ≥ 80% and all had minimal effectiveness (knockdown rate ≥ 75% or mortality rate ≥ 50%). In spite of the early drop below the bioassay cut-off values first generation PermaNet^® ^maintained a level of 15%–45% optimal and 30%–65% minimal effectiveness up to 27 months of follow up when rates appear to drop further suggesting that at least a proportion of these nets were functional as LLINs. Therefore, the distribution of knockdown and mortality values for first generation PermaNet^® ^at baseline and follow-up were further investigated. Between 6 and 27 months the distributions of results for knockdown as well as mortality showed clearly bi-modal curves, which were statistically different from baseline as well as from ITN at 6 months (p < 0.05 Kolmogorov-Smirnov test for equality of distributions).

**Table 4:** Net effectiveness based on WHO recommended criteria. Optimal effectiveness: KD60 ≥ 95% or mortality ≥ 80%, minimal effectiveness: KD60 ≥ 75% or mortality ≥ 50%.

**A**	**Proportion of nets with optimal effectiveness at different times of follow-up**					
**Type of net**	**Baseline (n) % *95% CI***	**6 Months (n) % *95% CI***	**12 Months (n) % *95% CI***	**18/20 Months (n) % *95% CI***	**24/27 Months (n) % *95% CI***	**36/39 Months (n) % *95% CI***

PermaNet 1^st ^generation	(10) **100** *69.2 – 100*	(40) **37.5** *22.7 – 54.2*	(40) **42.5** *27.0 – 59.1*	(40) **15.0** *5.7 – 29.8*	(40) **45.0** *29.3 – 61.5*	(38) **10.5** *2.9 – 24.8*
PermaNet 2^nd ^generation	(10) **100** *69.2 – 100*	(40) **100** *91.2 – 100*	(40) **97.5** *86.8 – 99.9*	(40) **97.5** *86.8 – 99.9*	(38) **71.0** *54.1 – 84.6*	(40) **90.0** *76.3 – 97.2*
Conventional net	(10) **100** *69.2 – 100*	(40) **7.5** *1.6 – 20.4*	Not done	Not done	Not done	Not done
Conventional net retreated	(13) **100** *75.3 – 100*	(34) **61.8** *43.6 – 77.6*	(11) **45.5** *16.8 – 76.6*	Not done	Not done	Not done

**B**	**Proportion of nets with minimal effectiveness at different times of follow-up**					

**Type of net**	**Baseline (n) % *95% CI***	**6 Months (n) % *95% CI***	**12 Months (n) % *95% CI***	**18/20 Months (n) % *95% CI***	**24/27 Months (n) % *95% CI***	**36/39 Months (n) % *95% CI***

PermaNet 1^st ^generation	(10) **100** *69.2 – 100*	(40) **70.0** *53.5 – 83.4*	(40) **60.0** *43.3 – 75.1*	(40) **30.0** *16.6 – 46.5*	(40) **65.0** *48.3 – 79.4*	(38) **18.4** *7.7 – 34.3*
PermaNet 2^nd ^generation	(10) **100** *69.2 – 100*	(40) **100** *91.2 – 100*	(40) **100** *91.2 – 100*	(40) **100** *91.2 – 100*	(38) **92.1** *78.6 – 98.3*	(40) **100** *91.2 – 100*
Conventional net	(10) **100** *69.2 – 100*	(40) **25.0** *12.7 – 41.2*	Not done	Not done	Not done	Not done
Conventional net retreated	(13) **100** *75.3 – 100*	(34) **73.5** *55.6 -87.1*	(11) **72.7** *39.0 – 99.9*	Not done	Not done	Not done

At baseline more than 1 sample was taken per net to allow assessment of within-net variation of insecticide concentration which was calculated as the mean difference from the mean for that net and expressed as % of the mean. For first and second generation PermaNet^® ^the within net variation was very similar with 18.2% and 16.8% (mean absolute difference to the net mean 7.2 mg/m^2 ^and 10.1 mg/m^2 ^respectively). In contrast, it was 110% for ITN. Between net variation of insecticide was calculated as the coefficient of variation (standard deviation expressed as % of mean) and was 31.5% for first generation PermaNet^®^, 20.6% for second generation and 96.9% for ITN.

**Figure 4:**
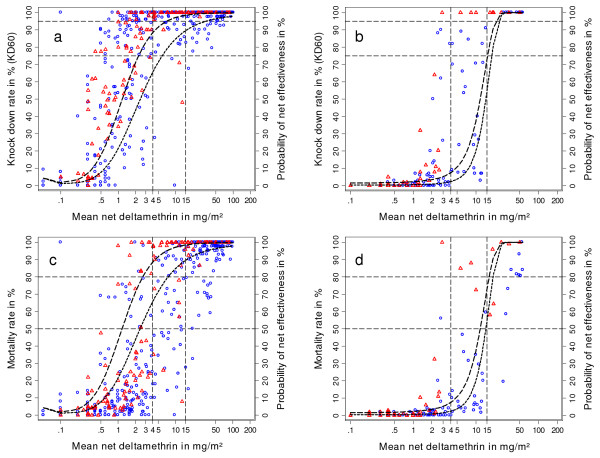
**Correlation between chemical and bioassay results**. Results for *Anopheles gambiae s.s*. panels a and c, *Culex quinquefasciatus *panels b and d. Red triangles represent conventionally treated nets, blue circles LLIN. Probability of minimal effectiveness (dashed line) and optimal effectiveness (dotted line) based on a probit model.

The correlation between chemical residue results on the one hand and bioassay and the probability of a net having optimal or minimal effectiveness (estimated from a probit model) on the other was explored for *Anopheles gambae s.s*. and *Culex quinquefasciatus *and the results are shown in Figure 4. Four observations can be made: first, the graphs show a significantly lower susceptibility of Culex to deltamethrin compared to Anopheles with values for knockdown rate and mortality sharply dropping at deltamethrin concentration between 20 mg/m^2 ^and 15 mg/m^2^: second, no principle difference in the correlation between chemical residue and bioassay results was observed between LLIN and ITN; third, for both species mortality rates began to drop early after only moderate decline of the insecticide concentration while knockdown rates remained high (> 75%) until a level of approximately 4 mg/m^2 ^was reached with Anopheles and 15 mg/m^2^for Culex. For Anopheles deltamethrin concentrations as low as 0.5 mg/m^2^were still frequently associated with knockdown rates above 75%; fourth, the probability of at least 90% that a net shows minimal or optimal effectiveness against Anopheles seems to correlate well with the cut-off levels of 4 mg/m^2^and 15 mg/m^2 ^respectively which then could be used as alternative tests for effectiveness. Out of 190 samples that did not fulfil the criteria for minimal effectiveness only 4 had a deltamethrin content of ≥ 4 mg/m^2^giving a specificity of such a test of 97.9% (95%CI 94.7–99.4). Since many samples fulfilling criteria for minimal effectiveness actually had deltamethrin concentrations below 4 mg/m^2 ^the sensitivity was not quite as high, 342 out of 454 true positives or 75.3% (71.1–79.2). Using deltamethrin ≥ 4 mg/m^2 ^to test for minimal effectiveness then provides a positive predictive value (ppv) > 99% in any setting where minimal effectiveness is 75% or higher and a ppv of > 95% if minimal effectiveness is 35% or more. Using ≥ 15 mg/m^2 ^deltamethrin on the net as test for optimal effectiveness produces similar results, specificity 96.1% (93.1–98.0), sensitivity 64.1% (59.0–69.0) and a ppv > 98% at occurrence of optimal effectiveness of 75% or more and ppv > 95% for optimal effectiveness 53% or more.

**Figure 5:**
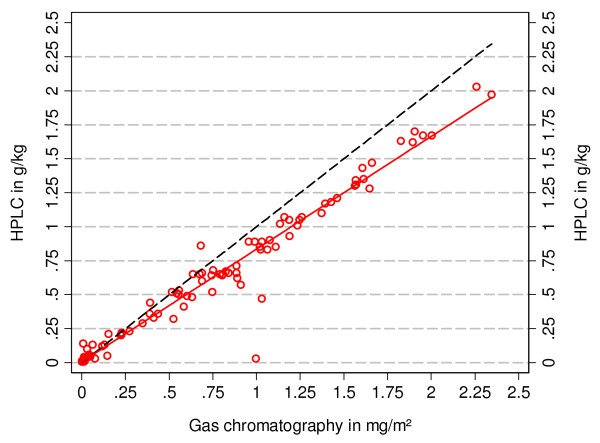
**Correlation between results from two analytical protocols for the determination of deltamethrin based on either gas chromatography (GC-ECD) or HPLC (CIPAC)**. The dotted (black) line represents equality between results and the continuous (red) line the linear regression.

During the study a total of 115 net samples were analysed for deltamethrin using different extraction and determination methods allowing a comparison. Figure 5 shows the correlation between the HPLC based method (#333 CIPAC) used by the Vestergaard-Frandsen quality control laboratories and the gas chromatography based method of the WHO collaborating centre (GC-ECD). While there was an excellent correlation between results (correlation coefficient 0.98, p < 0.00001) results from the CIPAC protocol were systematically lower than those from GC-ECD resulting in a difference of 0.30 g/kg at the level of 2.0 g/kg deltamethrin measured by gas chromatography.

In order to examine the potential loss of insecticide on the LLIN during long-term storage the 10 baseline nets of the first generation PermaNet^® ^were kept outside the package, exposed to light (but no direct sunlight) and dust at room temperature without ever being washed or used as mosquito nets. Roughly 1, 2, 3 and 5 years later samples were examined for chemical residue and 60 minute knockdown rate (*A. gambiae*) and results are shown in Table 5. Although there was some variation between time points the deltamethrin concentration had not noticeably declined (48.9 mg/m^2 ^after five years) nor was there a decline in the knockdown rate, 98% baseline, 93% after five years.

**Table 5:** Performance of stored nets: Chemical residue for PermaNet 1^st ^generation baseline samples unused, unwashed but exposed to air and light.

**Time**	**Deltamethrin on net**		**Bioassay results (A. gambiae)**	
In brackets samples/nets tested	Median (mg/m^2^)	Inter-Quartile-Range IQR (mg/m^2^)	Median Functional Mortality (%)	Inter-Quartile-Range IQR for mortality (%)

Baseline (40/10)	47.5	32.9 – 52.2	98.0	96 – 99
11 months (10/10)	46.4	37.4 – 49.3	99.0	85 – 100
27 months (10/10)	50.3	45.1 – 55.0	Not done	Not done
39 months (10/10)	42.9	34.9 – 47.6	97.0	96 – 98
60 months (10/10)	48.9	34.4 – 54.9	93.0	90 – 96

Finally, multivariate analysis was undertaken for the second generation LLIN to explore the relative role of washing and time of observation on insecticide level found on the net. A total of 197 samples were available for analysis varying in time of observation from 6 to 36 months but excluding the baseline nets as these were not distributed to households. Initially, socio-economic and demographic variables (wealth quintile, number of persons in household, education of head of household) were also tested but no associations were found and these variables were dropped. A linear regression model with deltamethrin concentration as dependent variable showed a decreasing trend for the insecticide with increasing washes (-2.26 mg/m^2 ^per wash, p = 0.015) and time (-0.98 mg/m^2 ^per month, p < 0.0001) and these variables together explained 27.8% of the variability of deltamethrin content (R-squared). Using fractional polynomial regression the relationship was explored to see whether more complex, non-linear functions would better fit the data but a linear relationship proved to be optimal. Running separate models for each year of observation suggested that the loss of insecticide per wash reduced over time with -5.2 mg/m^2 ^in the first, -2.9 in the second and -1.6 in the third year. Therefore, an interaction term between washing and time was introduced to test whether the change in rate of loss per wash over time was statistically significant, but this was not the case (p = 0.2). With a mean annual washing rate among these nets of 1.7 (95% CI 1.53–1.88) this implies that on average 3.8 mg/m^2^deltamethrin was lost per year through washing and 11.8 mg/m^2^per year through other factors somehow associated with use. Among the bioassay related outcomes only vector mortality and optimal effectiveness showed a significant association for time of observation (p = 0.01 and p = 0.03 respectively) but not number of washes.

## Discussion

Two possible approaches can be taken to study the field performance of a LLIN product under "real life" conditions. First, a cohort design can be used where a number of nets are randomly distributed to households and repeated measurements taken from the same net over time until a defined endpoint is reached. This design, which was applied by Lindblade and colleagues in Kenya [12], has the advantage that it follows the same net over time but the disadvantage that invasive measurements that require cutting of samples are limited to time of failure and bioassays have to be done on-site requiring an insectary within the study area. The second design option uses multiple random samples of nets with only one measurement per net. This approach which needs a larger number of nets but has no restrictions with respect to number of samples cut from each net for chemical and bioassay tests, is currently recommended by WHOPES [18] and has been applied in this study. By selecting the first generation PermaNet^® ^for the study randomly from a large batch of LLIN in the warehouse and distributing them randomly among households each random, cross-sectional sample of nets is representative of the original shipment of 10,000 nets to Uganda. In case of the second generation nets there was no large shipment to select from so that each time point represents the overall shipment from the factory. With the exception of the baseline nets only one sample per net was taken in a standardized fashion, i.e. from the same location on the net. This implies that the mean for all sampled nets per time point can be interpreted as a valid estimate of chemical residue, vector mortality or knockdown rate, while the result of an individual net sample has to be interpreted with caution as it will depend on the level of within-net variation of the insecticide and can not be taken as the true average of that net. To obtain an reliable estimate for a specific net a composite sample of at least 5 locations on the net has to be taken as described in the WHO specifications for deltamethrin LN [21] or better 12–14 as is practiced by the VF quality control laboratories (Phan, personal communication).

In the first generation PermaNet^® ^an unexpected drop in performance was observed after only 6 months at which time the rates for the outcome variables were not significantly different from conventionally treated ITN. However, thereafter chemical residue as well as bioassay results remained quite stable over more than 20 months with 15–45% of nets still showing optimal effectiveness. Even after 39 months of field use 5% of the net samples still had more than 4 mg/m^2 ^deltamethrin and 18% showed either a mosquito knockdown rate of ≥ 75% or mortality of ≥ 50% (minimal effectiveness). While this is not sufficient to fulfil WHOPES phase III criteria [18], it is clearly more than what can be expected from conventionally treated nets which have been reported to reach good performance up to 15 months [22] but never up to 27 months. The cause for the observed pattern of performance can not be explained by any of the variables in the data set, however, some potential causes can be excluded: excessive washing, use of more aggressive (alkaline) soaps and also the impact of sun light as none of the samples showed the R-isomer of deltamethrin typical for UV exposure (data not shown). The fact that these nets did not show any loss of activity even after 5 years when they were not used or washed (Table 5) and the bimodal distribution of results at the time points between 6 and 27 months suggest that a certain proportion of nets did loose the coating with the insecticide quickly under the stress of every day use and/or washing but the remaining 20% to 30% of nets continued to perform as LLIN at least up to 27 months and some up to 39 months. This explanation would also be consistent with the statement of the manufacturer that until early 2001 some problems in the manufacturing process existed that could have resulted in parts of a production batch having a lower quality and that between June 2000 and August 2002 continuous improvements on the production quality had been implemented (VF personal communication). This also implies that published data on the performance of the first generation PermaNet^® ^has to be seen in relation to the time of manufacture of tested nets. The first report on field evaluated PermaNet^® ^comes from Burkina Faso [10] where after 12 months mean delamethrin (n = 11) was 3.7 mg/m^2 ^with a mortality of 54% and after 18 months (n = 5) 1.6 mg/m^2^and 7% respectively. According to Kroeger and colleagues [23] the nets used in that study were an early version of the product manufactured in the first two months of 2000 while the PermaNet^® ^tested in Columbia by Kroeger and his team [9,20] were produced in the second part of 2000 at about the same time as the first generation nets used in this study. In the Columbia study six PermaNet^® ^were washed 20 times by local women during five months and then used by these families for another 2.5 years. After three years mean deltamethrin content (n = 4) was 9.6 mg/m^2 ^and mortality 88% [23]. Asidi *et al*. [24] tested one PermaNet^® ^in late 2000 after five washes using an experimental hut design and found no difference to conventionally treated nets. Similarly, Graham *et al *[13] reports on five wash and experimental hut trials of first generation PermaNet^® ^in three countries with nets being delivered between January 2000 and April 2002 and found mortality on the LLIN after 15 to 21 washes above 90% but median time to knockdown and blood feeding inhibition only significantly better than conventionally treated nets in one trial. In contrast, Gimnig and co workers [25] had good results in a field trial in Malawi using nets produced late 2001 with mortality 42.5% after 24 months of field use (n = 25) and 60% of the first generation PermaNet^® ^still having minimal effectiveness, very similar to results reported in this study. This varying performance seems to be in keeping with our hypothesis of inconsistent production quality of the nets between early 2000 and late 2001 with some batches or part of batches being better than others which in conjunction with the small sample sizes of the quoted studies would explain the variations in results. In contrast, tests on first generation PermaNet^® ^from first half of 2002 – just before the finalization of the second generation PermaNet^® ^(VF personal communication) – tested in a field trial in Kenya showed excellent performance after two years with mean mortality of 72% and 82% of the nets functional with mortality rates at all times greater than 50% [12]. These same nets washed in the laboratory 20 times under standard WHO conditions still showed 55% mortality, 72% knockdown and a chemical residue of 18.5 mg/m^2^, very close to what was found for the second generation PermaNet^® ^in this study.

To date there are only two studies published on the second generation PermaNet^® ^which was officially launched as PermaNet 2.0^® ^in April 2003. Both, however, were not phase III field trials but rather phase I/II washing trials. In the first study in Pakistan [13] PermaNet 2.0^® ^performed significantly better after 20 washes in the field than a conventional ITN with mortality 81.8%, knockdown 79.5% and a deltamenthrin residue of 24.1 mg/m^2 ^when net swatches were washed and 13.1 mg/m^2^when the whole net was washed. After 30 washes mortality dropped to 43.2% but knockdown rate remained high with 77.3%. Mean deltamethrin content at this time was 5.8 mg/m^2^. In the second study second generation PermaNet^® ^were used as a control in the laboratory study of another product [26]. Here a mortality rate of 100% was found after 30 washes with 18.7 mg/m^2 ^deltamethrin remaining on the net after these washes. Although these results are not directly comparable they indicate already the strong performance of the improved product. This is very similar to results from this study where only the 24 months sample showed slightly reduced figures for mortality (73.9%) and knockdown (92.4%), with a resulting proportion of nets with optimal effectiveness of 71.0 % (WHOPES phase III criteria 80%). However, the confidence interval for this sample did include the 80% mark and three of the nets had been washed immediately before collection which might have resulted in the poorer performance in the bioassay. Also, the deltamethrin content decline showed a continuous, more or less linear decline with no unusual drop at 24 months supporting the hypothesis that this lower value was a statistical outlier rather than a systematic decline in performance. At 36 months 90.0% (95% CI 76.3–97.2) of the second generation PermaNet^® ^had either mortality ≥ 80% or knockdown ≥ 95% and therefore fulfilled the WHOPES criteria for phase III LLIN evaluation [18]. The nets tested in this study were produced in July 2002, a few weeks before the finalization of the production details for the PermaNet 2.0^® ^brand but according to the manufacturer did not differ significantly from that product (VF personal communication).

The declared deltamethrin loading dose for the first generation PermaNet^® ^was 50 mg/m^2 ^and a mean of 43.6 mg/m^2 ^(95% CI 36.0–51.3) was found which compares well with other reported results of 44.9 [12], 47.1 [10], and 48.3 mg/m^2 ^[11]. In contrast, the baseline deltamethrin content for the second generation PermaNet^® ^was rather high with a mean of 67.1 mg/m^2 ^(95% CI 60.0–74.4), above the declared 55 mg/m^2^but still within the allowed upper limit of 68.7 mg/m^2^stated in the WHO specifications for deltamethrin on long-lasting (coated) insecticidal nets [21]. When the content was expressed in g/kg, the units of the actual measurement, the baseline value for the second generation PermaNet^® ^was 2.1 g/kg (95% CI 1.9–2.4) which is closer to the expected value of 1.8 g/kg and clearly within the required limits of 1.35–2.25 g/kg. This difference between mg/m^2 ^and g/kg results indicates that the actual, measured mass of net per unit was slightly higher than the 30 g/m^2 ^stated in the specifications and, in fact, it was found to be 31.7 g/m^2 ^from measurements taken according to the relevant ISO norm 3801 [27]. The range of deltamethrin content on the baseline nets was 52.5–80.4 mg/m^2 ^or 1.5–2.5 g/kg but since only two samples were taken per net this is a reflection of within net variability and – as outlined above – can not be interpreted as mean insecticide level for those nets. Other studies have also found rather high baseline insecticide values for the second generation PermaNet^®^. Yates et al. [26] using HPLC found a mean baseline deltamethrin concentration of 66.7 mg/m^2 ^and Graham *et al *[13] 86.3 mg/m^2 ^when they tested net swatches before washing but 55.3 mg/m^2 ^on the samples from nets prepared for washing.

Interestingly, a systematic difference was observed between results from the analytical methods used by the WHO collaboration centre (GC-ECD) and the VF quality control laboratories (HPLC-DAD, CIPAC) with the latter systematically lower than the former (Figure 5) and the difference reaching 0.30 g/kg at the 2.0 g/kg level. This would imply that a mean deltamethrin content of 2.1 g/kg found in the gas chromatography for the second generation PermaNet^® ^at baseline would correspond to 1.8 g/kg or 55 mg/m^2 ^in the methodology used by VF. It is concluded, therefore, that the baseline deltamethrin content of second generation PermaNet^® ^in this study was within the specified target dose with either method of determination. Furthermore, even if the initial deltamethrin loading had been slightly lower the result in the three year follow-up would not be significantly different as at 36 months on average well above 15 mg/m^2^deltamethrin would have remained on the nets which was shown to correlate with high bioassay results.

The difference between the analytical procedures was quite significant at higher deltamethrin content and needs further evaluation as both methods are frequently used in the testing of LLIN. It is not likely that the difference was caused by the fact that samples were not exactly identical although taken immediately next to each other on the net. Such a difference by intra-net variability of insecticide would be expected to go in both directions and would average very close to zero. A direct comparison of the two protocols, HPLC-DAD according to #333 CIPAC and GC-ECD according to ISO 17025 at the WHO Collaborating centre in Gembloux, Belgium (n = 11, two measurements per sample) by independent technicians showed almost the same linear relationship and systematic error at higher levels of insecticide as in our reported data with 2.0 g/kg in GC-ECD corresponding to 1.75 g/kg in HPLC-DAD (Pigeon, unpublished data). This means that the difference is also not caused by the execution of the protocol by either laboratory in our study but is a true, systematic difference in the methodologies. However, it is at this point not clear at which part of the protocol the deviation occurs: sample preparation, extraction or determination of insecticide content. Both analytical methods have been validated using samples with known deltamethrin content and shown to have a very high recovery rates of 99.7% (95% CI 98.6–101.6) in the case of the HPLC-DAD (CIPAC) method (Phan, unpublished data) and 98.2% (97.1–99.4) for the GC-ECD method (Pigeon, unpublished data). However, at a deltamethrin loading of more than 30 mg/m^2 ^recovery in the GC-ECD method was 101.2% (99.8–102.6). Repeated re-extracting of those samples consistently failed to show any remaining deltamethrin indicating that all insecticide was captured. The higher values measured in GC-ECD at high deltamethrin levels could possibly be explained by slight dilution variability. On the other hand, the sample preparation could also contribute to the systematic difference as in the HPLC-DAD (CIPAC) methodology the 10 × 10 cm sample is cut up into many small pieces before extraction which could lead to a mechanical loss of insecticide. The GC-ECD protocol is the more universally applicable method for insecticide determination in LLIN as it can be used for LLIN with coating as well as incorporation technology and is able to reliably detect even very small amounts of insecticide. The HPLC-DAD (CIPAC) protocol has the limitation that it can only be done with LLIN samples using coating technology but is very suitable for assessment of baseline loading dose of the LLIN and is, therefore, used by manufacturers for quality control purposes. As both methods will continue to play a significant role in the testing of new LLIN products the systematic difference described here clearly needs further study.

The initial batch of ITN also lost insecticide very quickly with a median deltamethrin content of only 0.7 mg/m^2 ^after six months of use and a vector mortality and knockdown rate of 8.5% and 31.6% respectively. Although reported results from deltamethrin treated conventional ITN after washing and/or field use vary considerably [11-13,23,25,28], this appears lower than expected. However, when the same nets were re-treated after 15 months they showed a chemical residue of 3.1 mg/m^2^, mortality of 40.8% and knockdown of 76.9% six months later and there was no difference to new polyester nets treated at the same time. These results are very similar to what was found by Gimnig in Malawi [25]. A possible explanation for the initial rapid loss could be the presence of some warping oil on the nets at the time of dipping or a poor quality of polyester that did not allow the insecticide to attach to the fiber. A second group of re-treated conventional nets had even better results with deltemethrin of 1.4 mg/m^2^, mortality 44.8% and knockdown 59.4% after 12 months. One possible explanation is a cumulative effect with insecticide remaining from the previous treatment [29] or a slightly better persistence of the deltamethrin tablets compared to the liquid formulation [28]. Nonetheless, these results are within the reported limits of performance as mortality of > 70% has been described with deltamethrin treated nets even after 15 months of fields use [22] and deltamethrin concentrations as low as 1.5 mg/m^2 ^[28].

Nets in our study were used regularly and washed mainly with cold water in a basin and without rubbing on stones, similar as has been described from Tanzania by Erlanger and colleagues [30]. With very few exceptions locally made soap bars were used rather than industrial detergents. The alkalinity of this soap was found to be moderate with ph 9–10 which has also been found in other studies [13,30] and can be considered favourable with respect to the potential to degrade deltamethrin compared to the industrial detergents. The washing frequency in our study was low with initially 2.2 washes per year and then declining in phase two to 1.8 washes per year. This is significantly lower than the 1.0 wash per month reported in Uganda from a descriptive household interview study [31]. However, as Miller *et al *have demonstrated in Tanzania reported washing does not correlate well with actually observed washes [32] the latter being significantly lower than the former. The washing frequency in our study areas was not that much different from some of the rates reported from other countries: Gambia 1.9 washes/year [28], Columbia 1.2 [23]. Other studies reported washing rates between 3.6 and 5.6 per year [5,12,30] but none in the range of 12 washes per year. It is difficult to say to which extent results on longevity of insecticidal effects observed in this study would have been different if the washing frequency would have been higher. But it might be useful for the WHOPES evaluation process to require studies in settings with differing washing habits.

In the washing study of PermaNet 2.0^® ^by Graham and colleagues [13] in Pakistan 72.1% of the baseline insecticide had been lost after 20 washes. This is slightly more than the 58.2% loss found in this study for second generation LLIN after three years. On the other hand, the loss was 59% after 20 washes in the laboratory washing study by Yates at al. [26]. This indicates that the loss observed in this study over three years would be equivalent to somewhere between 15 and 20 washes without field use. However, the mean number of washes actually observed for our tested nets was 4.5 with a range of 0–9. This suggests that under every day use in the field washing alone is not the only determinant of insecticide loss. This finding was further explored using a linear regression model with number of washes and time of field use as key variables and found that, indeed, more insecticide (75.6 %) was lost through every day use than by washing (24.4 %). It is not exactly clear whether physical handling of the nets or environmental factors or a mixture of these causes the loss and this issue will require further study. It is, however, quite clear from our results that at least for LLIN using coating technology, maximum number of washes obtained for a LLIN product in the laboratory without loss of functionality can not be translated into the same number of washes under field conditions. This emphasizes the need for phase III trials under field conditions in the process of WHOPES evaluation. The situation may be different for LLIN using incorporation technology [33].

A highly significant correlation was found between the chemical residue and bioassay results for samples taken from the same location on the net. A very similar relationship has been described by Yates and colleagues between chemical residue and median time to knockdown [26]. Adams et al. [34], on the other hand, did not find a statistically significant correlation between insecticide content and bioassay results possibly because none of the insecticide levels were below 3 mg/m^2 ^and most corresponding mortality values above 90% and all above 50%. At least for *Anopheles gambiae s.s*. deltamethrin levels as low as 1–3 mg/m^2 ^can produce knockdown rates between 75% and 95% and mortality above 50%, something that several authors have described previously [23,27,29,30,34]. *Culex quinquefasciatus *responded less sensitive to deltamethrin with bioassay results rapidly declining at insecticide levels below 15–20 mg/m^2^. This is thought to be due to a generally higher tolerance of this species to tarsal contact insecticides [22].

Based on a total of 578 observations, it could be established that using a cut-off of ≥ 4 mg/m^2 ^and ≥ 15 mg/m^2 ^deltamethrin on a net sample can distinguish nets with minimal and optimal performance in the bioassay with *An. gambiae *with a positive predictive value of > 95%. Sensitivity was rather low and this test, therefore, not suitable to identify an indivudual net that fails the effectiveness standards. But it would be possible to use chemical residue as a proxy to identify nets that continue to provide protection in situations where taking of large samples for bioassays is not possible. One must keep in mind, however, that this test would then need to assume that the insecticide found on or in the fibre also is bio-available to the mosquito which may not always be the case, particularly in LLIN that use the incorporation technology [11,12].

When investigating the longevity of protection one can expect from a LLIN product under programmatic conditions the duration of the insecticidal effect is only one part of the equation. The duration of the physical integrity of the netting material and the resulting useful life is equally important and in contrast to the former, there is very little published literature on the latter. Ritmeijer et al. [35] report 65% of fine-mesh, 75 denier polyester nets with any holes after two years in a leishmaniasis programme in Sudan and Spencer and co-workers [36] found 78% of 75 denier polyester nets with any holes 12–15 months after distribution in a refugee camp in western Uganda. Both these findings are within the results from this study (Figure 2). In a cross-sectional survey in Tanzania [30] 86% of polyester nets were found with any holes but the age range of the nets was 0.5–5 years and the fibre strength ranged from 40 to 100 denier.

Reports on the severity of damage are difficult to compare as each study used a different definition. They ranged from 45% severely damaged (> 7 holes larger 2 cm) in the cross-sectional net survey [30] to 33.2% with > 5 holes after two years in Sudan [35] to 28% with at least one hole of 40 cm^2 ^after 12–15 months in Uganda [36]. In order to provide a more standardized and continuous measure of the physical net condition a hole index was used which was constructed in analogy to the classical spleen index in malariology which combines the frequency of any hole with the size of these holes divided into three categories. It was demonstrated that the physical condition of the 75 denier polyester nets linearly deteriorated in the first 3 years but decay then seemed to accelerate somewhat with evidence that severely torn nets with a hole index of 30 or more were significantly more likely to be stored away rather than used.

Interestingly, the rate of hole acquisition (proportion with any hole) over time was slower in the second phase of the study after families had used nets already for two years. This trend continued in the current third phase of LLIN testing in the very same families where after 12 months only 34% of nets had any holes compared to 50% in phase two and 71% in phase one and after 18 months the figures were 44%, 64% and 80% respectively. This strongly suggests that net users learn to handle the net better or more carefully with increasing user experience and emphasizes the importance of a communication campaign to support the development of such a net culture.

While it is reasonably easy to monitor the physical condition of a net, it is very difficult to infer from these findings on the average useful life of a net under "real life" conditions as the continued use of a torn net will depend on many factors such as availability of a replacement, presence of other nets in the household that can be shared or perceived pressure to keep the net in a study or project setting. In this study 83.5% of the originally distributed polyester nets were still present after five years when those nets sampled or replaced at earlier times were not considered [see additional file 2]. This is similar to what has been reported for polyethylene nets in Tanzania where seven years after distribution 97% of households that received one or more nets still had at least one net in spite of the observation that 93% of the nets had any holes and 55% had 6–15 holes of 2 cm or more which would be equivalent to a hole index of 15–45 [33]. Although these polyethylene nets (equivalent to ~180 denier) are likely to be stronger than a 75 denier polyester net neither observation is likely to be equivalent to a realistic average useful life estimate due to the study/project situation. A household survey design may be a better tool to approach the useful life of nets and based on one such study is likely to average around 3 years for polyester nets of medium quality [37].

## Conclusion

Based on the evidence presented it is concluded that

1. The poor performance of a large proportion of first generation PermaNet^® ^was most likely due to inhomogeneous treatment at factory and not a failure of principle.

2. Second generation PermaNet^® ^performed well in this setting and fulfilled WHOPES phase III criteria.

3. Deltamethrin content of a net of more than 4 mg/m^2 ^or more than 15 mg/m^2 ^can be used as a proxy test for minimal and optimal effectiveness respectively with a positive predictive value of > 95% in most settings assuming the insecticide is also bio-available.

4. Loss of insecticide under field conditions in second generation PermaNet^® ^was not primarily a function of washing but rather of time which has to be seen as a proxy variable for regular use or handling of the net or exposure to environmental factors, although further research is needed to shed more light on which factors exactly play a role.

5. Polyester nets of 75 denier show serious signs of destruction after approximately three years in this poor, rural population and increase of the number of size of holes is then associated with less regular use.

6. There is a systematic difference between two commonly used analytical methods for the detection of deltamethrin in LLIN using the coating technology which at levels above 30 mg/m^2 ^are significant and need further clarification.

## Authors' contributions

AK developed the study concept and design, analysed and interpreted the data and drafted the manuscript; WB participated in the study design and data interpretation and coordinated all field work; OP carried out the chemical analysis using Gas Chromatography and contributed to interpretation of data; FA was responsible for bio assays during phase two of the study and contributed to data interpretation; SD was responsible for bio assays during phase one of the study and contributed to data interpretation; CP carried out chemical analysis using High Performance Liquid Chromatography and contributed to data interpretation. All authors contributed to finalization of the manuscript and read and approved the final version.

## Supplementary Material

Additional file 1 Table A. Timing and sample sizes of net follow-up surveys. Click here for file

Additional file 2 Table B. Number and times of collection of net samples. Click here for file
