# Supplementary material for: Long-term field performance of a polyester-based long-lasting insecticidal mosquito net in rural Uganda
Source: Malar J. 2008 Mar 20;7:49. doi: 10.1186/1475-2875-7-49 (PMC2330059; doi:10.1186/1475-2875-7-49)
Supplement: Additional file 1 — Table A. Timing and sample sizes of net follow-up surveys. [file 1475-2875-7-49-S1.pdf]

Table A: Timing and sample sizes of net follow-up surveys

Survey	Date	Total sample	Study round 1		Study round 2	
			# of nets surveyed	Months elapsed	# of nets surveyed	Months elapsed
0	23.-30.12.2000		*590 distributed*			
1	14.-17.02.2001	590	590	1.6		
2	12.-15.05.2001	590	590	4.5		
3	06.-12.10.2001	509	509	9.4		
4	21.-28.12.2001	505	505	11.8		
5	26.-30.04.2002	463	463	16.0		
6	01.-05.09.2002	442	442	20.1		
(0)	10.-15.10.2002				*260 distributed*	
7 (1)	07.-10.02.2003	466	210	25.4	256	3.8
8 (2)	13.-15.04.2003	452	194	27.5	258	6.0
9 (3)	02.-05.09.2003	355	140	32.1	215	10.6
10 (4)	07.-11.02.2004	301	128	37.4	174	15.8
11 (5)	22.-26.10.2004	215	90	45.8	125	24.2
12 (6)	12.-13.04.2005	159	78	51.4	81	29.8
13 (7)	11.-13.10.2005	152	81	57.3	71	35.8
