# Supplementary material for: Long-term field performance of a polyester-based long-lasting insecticidal mosquito net in rural Uganda
Source: Malar J. 2008 Mar 20;7:49. doi: 10.1186/1475-2875-7-49 (PMC2330059; doi:10.1186/1475-2875-7-49)
Supplement: Additional file 2 — Table B. Number and times of collection of net samples. [file 1475-2875-7-49-S2.pdf]

Table B: Collection of net samples. Figures in italic represent retreatments or exchanges of nets without removal. PN1= Permanet 1st generation, PN2=Permanet 2nd generation, conv=conventionally treated.

**Months since start**	**Time of net collection**	**Study nets sampled or removed**		
PN1 (PN2)		PN1 N=460	Conv N=150	PN2 N=270
0 --	Dec. 2000	10	10	
6 --	Jul. 2001	40	40	
12 --	Jan. 2002	40		
	Mar. 2002	*20 retreated*	*20 retreated, 40 exchanged*	
20 --	Sep. 2002	42 + 19 retr.	18 retr. + 34 exchanged	
-- (0)	Oct. 2002	144*	*39 retreated*	10
-- (3)	Jan. 2003		13 retr.	
27 (6)	Apr. 2003	40	14 retr.	40
-- (12)	Oct. 2003		11 retr.	40
39 (18)	Apr. 2004	38		40
-- (24)	Oct. 2004			38
-- (36)	Oct. 2005			40
	Total removed	373	140	208
	Seen last survey	71	10	35
	Lost to follow-up	16	0	27
